# A Scoping Review: The Impact of Housing Systems and Environmental Features on Beef Cattle Welfare

**DOI:** 10.3390/ani10040565

**Published:** 2020-03-27

**Authors:** Rachel M. Park, Margaret Foster, Courtney L. Daigle

**Affiliations:** 1 Department of Animal Science, Texas A&M University, College Station, TX 77845, USA; rmpark@ncsu.edu; 2 Medical Sciences Library, Texas A&M University, College Station, TX 77845, USA; margaretfoster@tamu.edu

**Keywords:** welfare, beef cattle, housing, floor type, space allowance, shade, environmental enrichment, ventilation

## Abstract

**Simple Summary:**

Reviews are needed to synthesize known information on a particular topic. Beef cattle welfare is an emergent research field. Decisions producers make, such as how to house cattle, can impact their overall welfare. Environmental features that can influence the microclimate and beef cattle welfare include floor type, space allowance, shade availability, and inclusion of enrichment (EE) devices or ventilation features. Therefore, the aim of this systematic review was to examine the relationship between housing and welfare metrics, so that beef cattle producers and animal scientists can make informed decisions regarding how their housing choices may impact beef cattle welfare. The databases searched were CAB (Ovid), AGRIS (Ovid), Agricola (EBSCO) and Searchable Proceedings of Animal Conferences. The final search was conducted on June 4, 2018. One reviewer determined relevancy of studies based on titles and abstracts. The full text was reviewed to determine study inclusion and four trained reviewers collected data from the included studies using pre-defined forms. From 1147 citations, 40 studies were included that evaluated the impact of a feature of beef cattle housing on welfare. In this review, we outlined how these features may positively or negatively impact cattle health, productivity, stress and behavior. Our main findings were as follows: (1) Space allowance influences feedlot cattle biology and behavior (2) Veal calves exhibited behavioral, physiological, and performance parameters indicative of a positive welfare state while group housed and (3) Provision of progressive modifications (e.g., shade, EE) to the feedlot environment resulted in increased performance of species-specific behaviors

**Abstract:**

Housing systems and environmental features can influence beef cattle welfare. To date, little information has been synthesized on this topic. The aim of this scoping review was to examine the relationship between housing and welfare status, so that beef cattle producers and animal scientists can make informed decisions regarding how their housing choices could impact beef cattle welfare. Housing features were categorized by floor type, space allowance and shade availability, as well as the inclusion of enrichment devices or ventilation features. Evaluation of space allowances across feedlot environments determined behavioral and production benefits when cattle were housed between 2.5 m^2^ to 3.0 m^2^ per animal. Over 19 different flooring types were investigated and across flooring types; straw flooring was viewed most favorably from a behavioral, production and hygiene standpoint. Veal calves experience enhanced welfare (e.g., improved behavioral, physiological, and performance metrics) when group housed. There is evidence that the implementation of progressive housing modifications (e.g., shade, environmental enrichment) could promote the behavioral welfare of feedlot cattle. This review presents the advantages and disadvantages of specific housing features on the welfare of beef cattle.

## 1. Introduction

Beef cattle housing varies based on stage of production, country, region, climate, and personal preferences of the producer. These factors ultimately lead to operation-specific management decisions (e.g., genetics, nutrition, weaning time, human-animal interactions, etc.). Veal calves, cow-calf operations, stockers and feedlots differ in their production goals, as well as the breed and age of cattle managed. These inherent differences among operations require that cattle housing vary based on the specific needs of the producer. Within the United States and across the globe, politics, legislation, social norms, and perceptions of beef cattle differ, thus impacting housing decisions made by producers. 

The environments that beef cattle will experience vary based upon geographical location, climate, sector of the production system, and these will influence the type of housing system used, i.e. the microclimate created [1,2], and therefore, the various available features within these housing systems (e.g., flooring type, space availability, shade access, enrichment and ventilation). Housing features create the environments and microclimates that cattle experience. The intensity, variation and type of environments created can have a direct impact on animal health, productivity and welfare. Therefore, evaluating how these housing differences impact the animal (e.g., behavior, physiology and productivity) will facilitate the understanding of the welfare status maintained within each specific system. 

Refereed reviews relative to beef cattle housing have been limited. The European Union Commission called for comprehensive reports and updates on the welfare of cattle maintained for beef production. This call has resulted in a total of three scientific opinion reports conducted by experts within the field, which cover a vast array of topics (e.g., housing systems, space allowance, castration, dehorning, genetics, nutrition, disease management) and their impact on beef cattle welfare. However, the reviews were limited to beef cattle production to countries within the European Union and were not peer-reviewed in nature [3,4,5]. Ingartsen and Anderson (1993) [6] examined the relationships among space allowance, housing type and flooring on the performance of cattle. Production measures examined included daily gain, feed intake, feed conversion, dressing percentages, carcass composition and conformation score. However, their review did not assess measures that may provide more insight into the welfare status of the animals under varying housing conditions, as the sole focus of that comparison was on performance metrics. To the authors’ knowledge, this is the first review that is systematic in nature that emphasizes the impact of all variations of housing (e.g., housing systems, flooring type, space allowance, shade availability, enrichment and ventilation) on beef cattle welfare and conducts comprehensive comparisons within each individual stage of production (e.g., finishing/fattening, cow-calf and veal calves).

A systematic analysis of scientific peer-reviewed literature is necessary to gain more insight into the relationship between beef cattle housing and the welfare of cattle reared for beef production. The review question agreed upon for this paper was “How do overall housing systems and housing facility features impact the status of animal welfare of beef cattle?” The primary purpose of this paper was to establish comparisons based on existing studies of the impacts that overall housing systems and housing features (e.g., flooring type, space availability, shade access, enrichment and ventilation) have on the welfare of cattle reared for beef production. While a multitude of factors influence animal welfare, this review will solely focus on housing impact and not discuss the additional impact of management decisions on beef cattle welfare, as that is beyond the scope of the included studies.

There were three main objectives of this review. The first aim of this review was to create a catalog of the housing systems, as well as the features of housing that producers have used throughout the various stages of beef production to characterize the advantages and disadvantages of the different housing options’ impact on beef cattle welfare. Secondly, the review was used to assemble measures that have been used to assess the welfare status of beef cattle in housing studies. Lastly, the relationships between housing and beef cattle welfare will be examined.

## 2. Materials and Methods 

This scoping review was conducted following the methodology outlined in O’Connor et al. (2014) [7] and Sargeant and O’Connor (2014) [8].

### 2.1. Eligibility Criteria

#### 2.1.1. Population and Interventions

The population considered for this scoping review consisted of beef cattle. Studies were required to examine housing as the intervention. Housing facilities were defined as pastures and/or buildings used to confine beef cattle for food production. Features of housing were defined as features of the housing units such as space allowance, flooring, shade, enrichment additions and/or ventilation.

#### 2.1.2. Comparators and Outcomes

All stages of production were included in an attempt to provide a comprehensive comparison of housing systems and housing features for producers and scientists, as well as highlight areas that need further investigation. Comparisons were made within, not among, production stages, e.g., veal calf studies were only compared to other veal calf studies, and so on. The outcomes investigated were the behavior, health, physiology, or production metrics reported in the study. Animal welfare was defined as how the animal is coping in the conditions in which it lives [9].

#### 2.1.3. Limitations

Studies were included from the following years: 1975 to 2018. Only papers written in English were considered for inclusion. 

### 2.2. Search

A librarian experienced in systematic reviews and animal literature searching designed the searches in four databases: CAB Abstracts (Ovid), AGRIS (Ovid), Agricola (Ebsco), and the Searchable Proceedings of Animal Conferences. Concepts included in the search were beef cattle, housing and welfare. Concepts were searched in keyword, thesaurus, title and abstract fields following the Cochrane Collaboration standards of search strategy structure. Searches were conducted between December 8, 2017 and April 11, 2018. Cab Abstracts was updated on June 4, 2018. See Table 1 for the details of the search. 

### 2.3. Selection

Citations were uploaded to Rayyan QRCI to be sorted for inclusion, on the basis of title and abstract content [10]. Inclusion keywords identified manually at this level included beef cattle and housing. Exclusion keywords identified manually, based on search results, included dairy, transport, reviews, rabbits, literature review, broiler, cross-sectional, age-matched, in vitro, cells, mice and fish. Only articles that conducted randomized control trials were included in this review. One reviewer (RMP) read all abstracts to identify potentially relevant studies. In the event, an abstract included an inclusion and exclusion word, the single reviewer (RMP) determined if the article should be moved to the next round of study assessment. These studies were then uploaded into RefWorks Proquest for full text to be acquired and reviewed. One reviewer (RMP) read all full articles to determine study inclusion in the scoping review. 

### 2.4. Coding and Appraisal 

A standardized form was used to extract data from studies that were determined to be relevant to the research topic. This form was designed to gather the following information: characteristics of the population, treatment details, features of the housing systems, types of measures recorded and outcomes. The significance level used for this paper was *p* < 0.05. In the event that a measurement was significant, the level of significance was recorded. Four reviewers extracted the data at this stage of the process. The extraction process was piloted by having all reviewers code one study. Results were discussed after to ensure accuracy in data extraction between all reviewers. The remaining studies were divided equally and randomly assigned to one reviewer to code. The Cochrane risk of bias tool, designed to determine the risk of bias in randomized controlled trials [10,11], was used to evaluate the methodological quality within the studies that were selected. A standardized form was created to assess each study. Two reviewers completed the form for each study. Reviewers assessed all studies, therefore, any disagreements in results were discussed between reviewers and a decision was determined that most accurately represented the study.

## 3. Results

### 3.1. Study Selection and Characteristics

**Figure 1:**
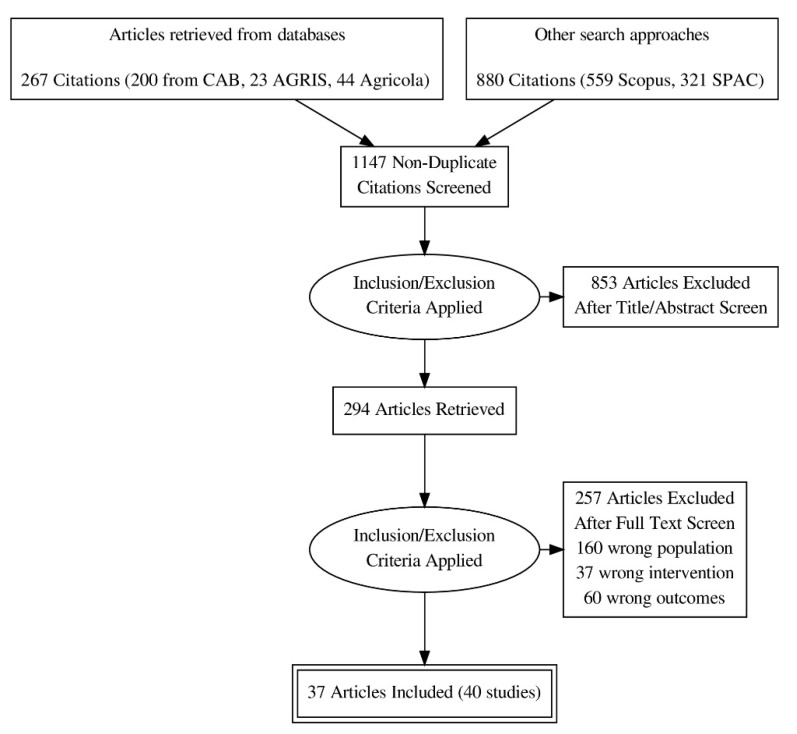
PRISMA flowchart depicting article inclusion of beef cattle housing studies measuring animal welfare criteria.

From the search, 267 citations were found from three different databases (CAB, AGRIS and Agricola), while 880 citations were found from other search approaches. In total, 1147 non-duplicate citations were screened for this scoping review. When inclusion and exclusion criteria were applied at the title and abstract level, 853 articles were excluded based on title and abstract content. For the remaining 294 articles, inclusion and exclusion criteria were applied following the completion of a full text screening process. A total of 257 articles were excluded due to wrong population, wrong intervention or wrong outcomes. Therefore, after conducting search and study selection, 40 studies were selected for inclusion. See PRISMA flowchart for numbers (Figure 1). 

Studies spanned across five different continents, with the majority conducted in Europe (26 studies) and North America (eight studies), followed by Asia (three studies), Africa (two studies) and Australia (one study). Experimental length of studies ranged from 22 days to two years, with two studies not providing their timeline. The majority of studies evaluated focused on the fattening stage (37 studies) with the remainder concentrating on veal calves (two studies) and cow-calf (one study). These studies housed cattle in the following systems: feedlot pens (34 studies), pasture (six studies), barns (four studies) or crates/pens (two studies). Studies were grouped into what housing feature they evaluated, which included the following: floor type (17 studies), housing system (eight studies), shade (eight studies), space allowance (six studies) or miscellaneous (four studies; enrichment—two studies, roofing and ventilation).

The number of animals used ranged from 8 to 2700, with the average number of animals used being 318. Over 30 breeds of cattle were represented, with eleven studies evaluating cattle that had a Charolais influence. Animals ranged in age from < one year to seven years, the majority of studies evaluated animals < one year of age, and 18 studies did not report the age of cattle used. The majority of studies evaluated bulls (17 studies) or steers (14 studies) followed by heifers (14 studies), veal bull calves (two studies) and cows (one study; Table S1). 

### 3.2. Measures

Over 232 various measures were recorded across the 40 studies, which included 82 behavioral measures, 31 health measures, 67 physiological measures and 52 production measures. The measures discussed below were identified by the author (RMP) of the scoping review as pertinent to beef cattle welfare evaluation with regards to housing (Table 2). Due to the number of measures, only those with significant results were included in Tables 4–7 and Table S2. Three studies did not have significant findings, or presented findings in a manner that results were not able to be synthesized and therefore will not be discussed further in this review [12,13,14]. 

### 3.3. Housing Systems

Overall, eleven different housing systems were evaluated from eight separate studies. Definitions of overall housing systems are provided in Table 3. See Table 4 for comparison of overall housing systems treatments and their impacts on beef cattle welfare. Cattle housed in tie-stalls had reduced welfare compared to those in loose housing, as is reflected in the greater concentrations of physiological indicators. Veal calves experienced negative behavioral, physiological, and performance consequences when housed in individual wooden crates, compared to group pens. Significant findings indicated detrimental impacts on cattle behavior for animals housed in a confined feedlot, compared to having access to pasture or being raised in pasture, as well as in comparison to cattle housed in a hoop barn. However, housing cattle on pasture, compared to a feedlot, may have a mixed impact on welfare, as demonstrated by changes in physiological indicators. Compared to pasture-housed cattle, cattle housed in a loose barn compared to pasture excelled in some areas of welfare, as indicated through performance and physiological based measures, however, had mixed behavioral responses.

### 3.4. Space Allowance and Flooring 

A comparison of space allowance demonstrated that smaller space allowances can negatively impact welfare (e.g., decrease in lying, ruminating, and positive social behavior, reduced feed efficiency and productivity, greater concentrations of stress hormones), whereas larger space allowances can positively impact welfare (e.g., reduced frequency of abnormal behaviors, greater body weights, and lower stress hormone concentrations). There is an exception to this when examining cattle provided 3.0m^2^ per animal, compared to those provided 1.5m^2^ per animal, as the responses observed suggest that the impact of space allocation on cattle welfare was mixed (Table 5). Across the six studies that investigated space allowance, the average initial live weight of animals ranged from 466 kg to 590 kg, with the exception of the study conducted by Ruis-Heutinck et al. (2000) [22], where cattle were enrolled with an average initial live weight of 217 kg and average final live weight ranged from 527 kg to 705 kg. One study [23] did not report final live weight of animals and is not included in the average final live weight range. Only one study [24] reported the average stocking density for each space allowance treatment, therefore this review can only compare space allowances of studies as expressed by m^2^ per animal. In total, 17 studies examined flooring type as a housing feature for beef cattle. Across these studies, 19 different flooring types were evaluated (Table S2). Fourteen of these flooring types were examined in only one study each. A fully slatted concrete floor was the most examined flooring option and was compared to 12 different flooring types across 11 different studies. Fully slatted rubber flooring and deep litter were the next most examined flooring options, being used in four different studies, respectively.

### 3.5. Shade and Miscellaneous Housing Features

Studies evaluating shade were examined to determine the benefits and drawbacks of this intervention (Table 6). Shade had a positive impact on beef cattle welfare as reflected in the behavioral, physiological and performance indicators reported. The remaining studies varied in which housing features were evaluated, including enrichment devices, roofing types and ventilation. Primarily these interventions had positive or neutral impacts on the cattle studied. For example, when tested for preference, cattle provided a brush interacted with this type of enrichment the most frequently and for the longest duration of time (Table 7). The presence of enrichment had no observed negative impacts on cattle health or performance. 

### 3.6. Cochrane Risk of Bias

**Figure 2:**
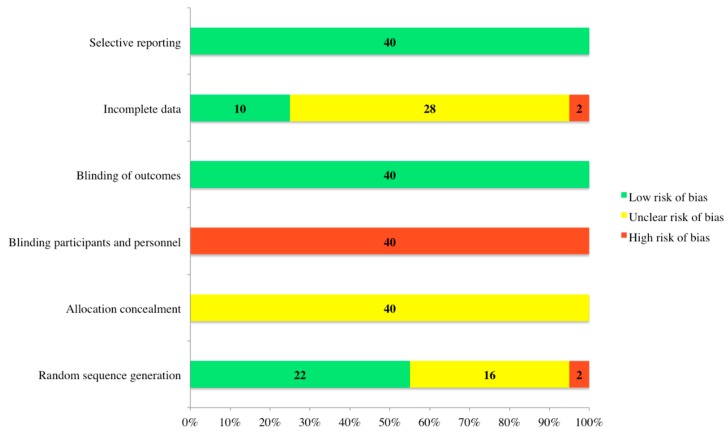
Results of the Cochrane risk of bias analysis conducted on all studies by two trained observers.

Only randomized controlled trials were reviewed to assess for risk of bias. All studies were evaluated utilizing the Cochrane risk of bias tool by two researchers [11,37] (Figure 2). No studies were removed from the review due to their results from the Cochrane risk of bias analysis. All beef cattle housing studies selected evaluated a comprehensive suite of welfare metrics, as well as ensuring that animals assigned to the control treatment are assessed on the same outcomes as animals who were provided the treatment(s). However, these same studies failed to blind the animal caretakers to the treatment assignment. In all studies, it was unclear whether the person enrolling cattle into treatments was aware of the allocation sequence. Studies varied with regard to how animals were randomly allocated to treatments and whether there were deviations in data due to removal of animals from specific treatment groups.

## 4. Discussion

### 4.1. Summary of Main Findings 

Housing systems vary within the beef cattle industry by stage of production and production outcome. Loose barn housing, compared to pasture housing, presents advantages and disadvantages to cattle welfare. Cattle housed in the loose barn had greater final live weights, ADG and BCS [17]. Loose barn housed cattle also performed fewer mounting events [15], spent less time vocalizing [17], spent less time walking [15,17] and spent more time engaged in lying behavior [15]. However, loose barn housed cattle spent more time standing [17], engaging in agonistic interactions [17], and performing oral explorative and oral manipulative behaviors [15,17]. Stravaggi Cucuzza et al. (2014) [21] conducted a study to compare loose housing to tie-stall housing. His research group demonstrated that tie-stall housing was stressful to cattle in the fattening stage, as animals housed in a tie-stall barn had greater levels of total serum protein, serum lysozymes, fecal corticosterone, serum corticosterone and cortisol. From these findings, loose housing was considered more favorable in comparison to tie-stall housing. 

Studies examining feedlot housed cattle observed that cattle in feedlots engaged in agonistic behaviors more frequently and for longer durations (e.g., headbutting, pushing, displacement) [19] compared to cattle with access to pasture. Similarly, feedlot-housed cattle spent more time standing and walking, as well as engaged in lying for a shorter duration of time paralleled to cattle housed in a hoop barn [18]. Environmental enrichment may be an effective environmental intervention that is designed to change feedlot cattle behavior, as this may increase the diversity and appropriately distribute the cattle’s behavioral repertoire. The intervention of environmental enrichment will be discussed in further detail later on in this review. Although studies were limited that examined veal calf housing, the findings provided overwhelming support for group housing compared to individual crates. Housing veal calves in groups resulted in a greater expression of social behaviors [16], a reduced expression of stereotypic behaviors [20] and improved carcass traits [20]. 

Cattle can benefit from an increased space allowance in the feedlot. Feedlot environments that provided animals with 3.0m^2^ to 4.5m^2^ per animal had greater live weight gains [25], as well as greater ADG [27] and lower feed conversion rates [23]. These animals performed a greater amount of positive social behaviors [25], spent a higher percentage of their day lying [22] and performed fewer abnormal behaviors [22]. However, Fisher et al. (1997) [25] found that cattle housed at a space allowance of 3.0 m^2^ per animal, compared to those in an environment of 1.5m^2^ per animal, had a greater mean- and peak-ACTH cortisol concentration. The authors of that study hypothesized that animals housed in the 1.5 m^2^ per animal housing were restricted in movement, and therefore exposed to chronic overcrowding, which may have resulted in adrenal fatigue (e.g., a reduction of responsiveness in the adrenal gland to ACTH). Overall, feedlots that provided cattle with 1.5 m^2^ per animal fared the poorest. Cattle in this setting spent less time lying [23,24,25], indicating a decreased comfort state, as this behavior is an indicator of cattle comfort and animal managers have a goal of promoting lying as part of good husbandry and to promote productivity. Hickey et al. (2003) [23] determined that highly stocked cattle did not interact socially as often as cattle with greater space allowances. High stocking density also had a negative impact on productivity and performance. Cattle that were provided 1.5 m^2^ per animal had reduced ADG [26,27] and final body weights [26], and also had higher feed conversion ratios [23]. However, animals at this space allowance did have greater kill-out proportions, or ratios of carcass weight to live weight [25,26]. The findings from these studies indicate that the difference between providing 2.5 m^2^ per animal to 3.0 m^2^ per animal could be substantial regarding the improvement of cattle welfare. However, there is not a clear understanding as to when increasing space allowances no longer provides additional benefits. Furthermore, future researchers investigating stocking density should consider providing the average space allowance in final weight per m^2^ for all treatments, as this could allow for more thorough comparisons of results moving forward.

Rearing cattle for fattening in a feedlot requires consideration of how flooring surfaces impact cattle welfare. Concerns have been raised regarding the use of fully slatted concrete floors, as this flooring type has been viewed as suboptimal for the animals’ welfare needs, particularly with regards to incidence of injuries [1,2]. This claim is partially supported by the findings of this scoping review. Cattle housed on fully slatted concrete floors performed greater frequencies of abnormal behaviors [22], had more unsuccessful lying attempts [38] and had a higher prevalence of health issues (e.g., skin lesions, locomotor disorders) [38,39] in comparison to fully slatted rubber mats. Fully slatted rubber mats resulted in greater live weight gains [38,40], ADG [38,40], feed conversions [40] and fewer health issues [22,41]. However, cattle housed on fully slatted rubber mats performed more agonistic behaviors compared to those on fully slatted concrete floors, which may be attributed to the animal’s pain status, as cattle in less pain possess the resources to engage in behaviors needed to establish and maintain a social structure [38]. Animals housed on fully slatted concrete floors, as well as animals housed on fully slatted rubber mats, displayed mixed results in comparison to specific mat conditions (e.g., foam structure rubber, natural rubber structure, partial cover of a solid mat, etc.), displaying both welfare advantages and disadvantages as indicated through behavioral, performance and health measures. For further detail, see Table 6. Cattle housed on straw had a greater frequency of lying behavior [22], improved hygiene scores [42] and enhanced performance measures (e.g., improved feed conversion ratio, higher ADG, greater carcass weight) [27]. These results suggest that cattle housed on straw floors had an enhanced welfare state compared to those housed on flat concrete, fully slatted concrete, or fully slatted rubber mats. This review highlights that there are advantages and disadvantages to all evaluated flooring types. 

The benefits of implementing shade outweigh any possible negative impacts, and the findings from this review strongly support the implementation of shade in the feedlot setting. Access to shade allows cattle to have a choice to reduce thermal stress, in a manner that does not compromise their performance or welfare. Cattle housed in an environment with shade have lower respiration rates [31,32], and lower panting scores [28,29] compared to their counterparts without shade. Animals provided shade were more willing to eat, as shade reduced the impact of temperature highs during the middle of the day [28]. Cattle with access to shade had numerous performance benefits, as well, including greater final body weights [28,29,31], ADG [29], DMI [29,30,31] and G:F [29]. The sole negative impact found of shade implementation was in conflict with another study. Gaughan et al. (2010) [29] found that shaded cattle had a lower dressing percentage, in contrast to Hagenmaier et al. (2016) [30], that showed that cattle in an environment with shade had greater dressing percentages. Therefore, the impact of shade on dressing percentage is unclear. As the EFSA Scientific Report (2012) [2] recommended beef producers that housed cattle in confined houses or open feedlots implement structures to reduce the effects of thermal stress, the studies reviewed here demonstrate that providing shade could be a realistic solution, as overall, the listed benefits of shade outweigh the possible negative impacts, which were limited.

Inclusion of environmental enrichment in beef cattle housing systems may be the next step to advancing cattle welfare, as well as promoting a positive public perception of beef cattle production. Few studies were found that evaluated the impact of environmental enrichment on beef cattle, which is reflective of the scarcity of current literature available on the topic. However, the two studies that were evaluated demonstrated that environmental enrichment can have either a positive or neutral impact on cattle welfare. Ninomiya and Sato (2009) [34] provided feedlot steers with a log and brush and found that steers with access to these items spent a greater percentage of time eating, yet no positive impact on productivity was observed. In another study, the impact of a grooming device was compared to different scent releasing (blank—no scent, lavender and milk) devices on feedlot heifers. Heifers interacted most frequently and for the longest duration of time with the rubbing device, followed by the milk-scent releasing device [35]. The findings of this review suggest that, within a feedlot setting, environmental enrichment that allows animals to perform grooming behaviors may be biologically appropriate, as this is a behavior that cattle are highly motivated to perform and cattle willingly and regularly interact with a brush. Further research is needed to evaluate the long-term welfare consequences of environmental enrichment in all stages of beef cattle production. 

This scoping review succeeded in investigating multiple research databases to gather the greatest number of studies related to the topic. More notably, the author (RMP) took additional approaches to review studies, by examining all the articles that were cited by accepted studies, as well as articles that cited the accepted studies. The consultation between the author (RMP) and a systematic review librarian (MF) was the greatest strength of this review. The National Academies of Sciences, Engineering and Medicine standard for systematic reviews calls for searches to be designed by information specialists and a recent study has shown that searches designed by librarians for systematic reviews improve the quality of reviews [43,44]. As the review was restricted to randomized controlled trials, there was the opportunity to assess the risk of bias for each individual study, which was viewed to be both an advantage and disadvantage. Conducting the risk of bias allowed for a more thorough analysis of these studies from a methodological standpoint, and it also assisted in determining features where beef cattle housing studies need improvement. However, this limited the review to only assessing randomized controlled trials, therefore excluding housing studies that did not fit the criteria of a randomized control design, which may have differing results that were not taken into account. 

Conducting the Cochrane risk of bias analysis demonstrated that researchers in this field running randomized controlled trials are doing well in reporting results on all the measures obtained, as well as assessing animals on the same measures, regardless of what treatment they were allocated to. However, this analysis also determined areas in which beef cattle housing randomized controlled trials could improve. A statement of the random allocation of animals to treatment groups is inherent to randomized controlled trials and must be included in the communication of this research. There cannot be an assumption that readers will know that random allocation occurred. Additionally, researchers need to ensure the reader that there is not a deviation of data in the results due to the removal of animals from specific treatment groups. There was a lack of clarity in the majority of studies evaluated, as to whether animals were removed or not and if animals were removed, and no reference was made to how their removal impacted the study. Areas of the Cochrane risk of bias analysis that beef cattle housing studies did not excel in, and are not likely to improve in, include both allocation concealment and blinding of participants and personnel. There was a consensus in that no studies reported on whether the person enrolling cattle into the treatment had knowledge of the treatment allocation. In randomized controlled trials, knowledge of treatment allocation is considered to be selection bias. However, ensuring that the person enrolling cattle in treatments does not know of which treatment the animal are entering into would be difficult, due to the impossibility of blinding personnel to the treatments. In housing studies, the interventions were apparent, e.g., a clear distinction between fully slatted concrete flooring and deep litter. The differences of treatments were visual and obvious. While researchers were not able to change this concept in most housing studies, effort should be made to ensure readers understand the reasoning behind a non-blinded study, as well as knowledge of treatment allocation.

### 4.2. Limitations

From an evaluation standpoint, comparing results across studies was not possible, as individual studies differed greatly in the measures that they assessed. This review highlights that there is not a consensus among researchers of what measures should be assessed when evaluating the impact of housing on beef cattle welfare. This is an area that requires improvement from researchers, to determine how to efficiently measure the animal’s response to housing. Measuring the response on similar scales will aid in future comparison of research, leading to more thorough conclusions regarding the impact of housing systems and their features on the welfare of beef cattle. Including a justification for why a metric was included for evaluation of welfare will assist in this effort. In addition to varying in measures, studies also varied in the housing evaluated. The majority of studies that were found and reviewed examined feedlot housing relative to the finishing stage. Therefore, this review was restricted in its ability to cover all stages of beef production due to the lack of studies in this area. Research that examined how housing impacts calves and cows in all stages of production was lacking. Further research is needed to conclude what the impacts of housing systems and features are on the welfare of these animals. 

## 5. Conclusions

Beef cattle producers need to understand how the housing decisions they make impact the performance, health status, physiology, and behavioral repertoire of the cattle in their care. Optimal space allowance for finishing animals may be between 2.5 m^2^ to 3.0 m^2^ per animal, while 1.5 m^2^ per animal may not be in the animal’s best interest, as animals housed at this space allowance had compromised productivity and performed behaviors reflective of a poor welfare state. The different flooring types investigated demonstrated positive and negative impacts on welfare; however, straw bedded flooring was associated with combination of behavioral, production and hygiene metrics that suggest this flooring has a positive impact on cattle welfare. Veal calves housed individually, compared to those housed in groups, demonstrated behavioral, physiological and performance metrics indicative of poor welfare. From this review, there is evidence that implementing progressive modifications to the feedlot cattle’s environment (e.g., providing shade or environmental enrichment) can positively impact cattle welfare. Economics ultimately influence final housing and environmental management decisions. However, the results from this review provide information regarding the long-term management, product quality, and economic consequences of different housing environments on beef cattle welfare that can be used to facilitate current and future feedlot cattle management strategies.

## Figures and Tables

**Table 1:** CAB Abstracts (Ovid) search details.

Search Order	Search Terms
1.	exp beef cattle/
2.	(beef adj2 (cattle or cow*or bull)).ti,ab.
3.	or/1–2
4.	exp calf housing/ or exp housing/ or exp cattle housing/
5.	(housing or barn* or pasture* or hill* or feedlot*).ti,ab.
6.	or/4–5
8.	exp animal welfare/
9.	(welfare* or wellbeing).ti,ab.
10.	or/9–10
11.	3 and 6 and 10
12.	limit 11 to English language

This search combined the concepts of cattle, housing, and welfare together. Lines 1, 4, 8 detail the CAB thesaurus terms searched for each concept. Line 2,5,9 detail terms searched in the title/abstract fields for each concept. Lines 3,6,10 show how these were OR’d together. Line 11 brings all of the concepts together. The asterisk is used to retrieve multiple endings to words. For example, cow* will return cow or cows.

**Table 2:** Author-selected behavioral, health, physiological, and production measures that were extracted from the included beef cattle housing studies. The number beside each measure indicates how many studies in this scoping review reported that specific metric.

Behavior	Health	Physiology	Production
Eating—23	Hygiene scores—13	Hemoglobin—7	Live weight—29
Lying—22	Lesions / swellings—7	Neutrophil—6	Average Daily Gain (ADG)—19
Standing—21	Hoof lesions—6	Red blood cell—6	Feed efficiency ^a^—12
Allogrooming—16	Hairless patches—5	Cortisol—5	Carcass external fat ^b^—12
Headbutt—13	Body Condition Score (BCS)—4	Lymphocyte—5	Dry Matter Intake (DMI)—11
Self-grooming—13	Bursitis—4	Platelet—5	Carcass conformation score ^c^—10
Mounting—12	Lameness score—4	Basophil—4	Carcass fat score—9
Drinking—11	% culls—4	Eosinophil—4	Carcass internal fat ^d^—9
Ruminating—11	Panting score—3	Fibrinogen—4	Carcass weight—9
Agonistic / Aggression—6	Nasal discharge—3	Haptoglobin—4	Dressing %—8
Walking—6	Abnormal breathing—1	Hematocrit (%)—4	Kill-out proportion—7
Inactive—5	Abrasions—1	Leukocyte—4	Marbling score—5
Tongue rolling—5	Coughing—1	Monocyte—4	Hot Carcass Weight (HCW)—4
Utilizing shade—5	Joint swelling—1		Water intake—3
Intentions to lie down—4	Ocular discharge—1		
Licking / manipulating objects—4	Mortality (%)—1		
Slipping—4	Treatments (%)—1		
Avoidance Distance at Feedrack (ADF)—3			
Abnormal lying sequence—2			
Displacement—2			
Interaction with enrichment—2			
Grazing—2			
Temperament score—1			

^a^ Feed efficiency includes feed conversion ratio and F:G. ^b^ Carcass external fat includes carcass fat score, fat thickness, mean subcutaneous fat depth, P8 fat, rib fat, 12th rib fat depth. ^c^ Carcass conformation score includes USDA yield grade and EUROP class scale. ^d^ Carcass internal fat includes kidney and channel fat weights, percentage kidney, pelvic and heart fat, perinephric and retroperitoneal fat.

**Table 3:** Overall housing system definitions used by systematic review authors. These are not equivalent to housing systems’ treatments, which are defined by the original study authors, but are used to describe the housing systems throughout the systematic review.

Overall Housing System	Definition
Feedlot	A pen that provides a predefined area of space, where cattle can move freely throughout the pen. Can be indoors or outdoors.
Hoop barn	A structure consisting of steel arches fastened to wooden side walls, covered with a UV-resistant polyvinyl tarp.
Loose housing / barn	An open barn, with a dedicated lying area, where cattle can move freely throughout the structure.
Pasture	A predefined area of land that houses cattle and provides suitable forage for grazing.
Tie stalls	Animal is tethered to a specific stall within a barn.

**Table 4:** Overview of the effect of different housing system types on beef cattle behavior, productivity, product quality and physiology. Housing systems are reported in the same language as they were presented by original study authors. Inclusion of significant results was determined at *p* < 0.05.

Metric Evaluated	Housing System					Reference, Location
	Confined Feedlot (CF)	Loose Barn (LB)	Feedlot with Shelter (FS)	Pasture (P)	Individual Wooden Crates (I)	
**Behavior**						
Allogrooming						
Duration	-	<P	-	-	-	[15] ^g^, Finland
Frequency	-	-	-	-	<GP ^a^	[16] ^f^, Italy
Feeding						
Duration	-	>P	-	-	-	[17] ^f^, [15] ^g^, Italy, Finland
Foraging						
Duration	-	<P	-	-	-	[15] ^g^, Finland
Lying						
Duration	-	<P	<HB ^b^	-	-	[18] ^e^, [15] ^f^, USA, Finland
Mounting						
Duration	-	<P	-	-	-	[15] ^g^, Finland
Negative social behaviors	>CFP ^c^ and P	>P		-	-	[19] ^f^, Uruguay
Rumination						
Duration	-	<P	-	-	-	[15] ^g^, Finland
Self-grooming						
Frequency	-	-	-	-	>GP	[20] ^f^, Italy
Sham rumination						
Frequency	-	-	-	-	<GP	[20] ^f^, Italy
Standing						
Duration	-	>P	>HB	-	-	[18] ^e^, [15] ^f^, USA, Finland
Tongue play						
Frequency	-	-	-	-	>GP	[20] ^f^, Italy
Vocalization						
Duration	-	<P	-	-	-	[17] ^f^, Italy
Waking						
Duration	-	<P	>HB	-	-	[18] ^e^, [15] ^f^, USA, Finland
**Productivity**						
ADG	-	>P	-	-	-	[17] ^f^, Italy
BCS	-	>P	-	-	-	[17] ^f^, Italy
Final live weight	-	>P	-	-	-	[17] ^f^, Italy
**Product quality**						
Color scores	-	-	-	-	<GP	[20] ^f^, Italy
Cooking weight loss (%)	-	-	-	-	>GP	[20] ^f^, Italy
EUROP scores	-	-	-	-	<GP	[20] ^f^, Italy
Flavor score	-	-	-	-	<GP	[20] ^f^, Italy
Tenderness score	-	-	-	-	<GP	[20] ^f^, Italy
**Physiology**						
Alkaline phosphate	-	-	-	<CF and CFP	-	[19] ^f^, Uruguay
Blood urea nitrogen levels	-	-	-	>CF and CFP	-	[19] ^f^, Uruguay
Calcium levels	-	-	-	-	-	[17] ^f^, [15] ^g^, Italy, Finland
Cortisol (fecal, serum)	-	<TS ^d^	-	-	-	[21] ^f^, Italy
Hemoglobin	-	-	-	-	<GP	[20] ^f^, Italy
Lysozyme	-	<TS	-	-	-	[21] ^f^, Italy
Packed cell volume (%)	-	-	-	-	<GP	[20] ^f^, Italy
Serum protein	-	<TS	-	-	-	[21] ^f^, Italy

^a^ Group pens (GP); ^b^ Hoop barn (HB); ^c^ Confined feedlot with access to pasture (CFP); ^d^ Tie stalls (TS); *^e^* Age not provided; ^f^ < 1 year; ^g^ 1–2 years.

**Table 5:** Overview of the effect of different space allowances on beef cattle behavior, productivity and physiology. Space allowances evaluated include 1.5, 2.0, 2.5, 3.0, 4.0, 4.2, 4.5 and 6.0. Space allowances are reported in the same language as they were presented by original study authors. Inclusion of significant results was determined at *p* < 0.05.

Metric Evaluated	Space Allowance (m2/animal)				Reference, Location
	1.5	2.0	3.0	4.5	
**Behavior**					
Abnormal behavior					
Frequency	-	>4.2	-	-	[22] ^b^, Netherlands
Eating					
Duration	-	-	<1.5 and 2.5	-	[23] ^s^, Ireland
Lying					
Duration	<2.0, 2.5, 3.0 and 4.0	<2.5 and 3.0	-	-	[23] ^a^, [25] ^a^, [26] ^a^, [24] ^c^, Ireland
Proportion	-	<4.2	-	-	[22] ^b^, Netherlands
Positive social interactions	<3.0 and 4.0	-	-	-	[23] ^a^, [25] ^a^, Ireland
Rumination					
Duration	<2.0 and 3.0	-	-	-	[26] ^a^, Ireland
Self-grooming					
Proportion	-	<2.5 and 3.0	-	-	[24] ^c^, Ireland
**Productivity**					
ADG	<2.0, 2.5 and 3.0	<2.5 and 3.0	-	>3.0 and 6.0	[26] ^a^, [24] ^c^, [27] ^c^, Ireland
Carcass weight	<2.5, 3.0 and 4.0	<2.5 and 3.0	-	-	[23] ^a^, [25] ^a^, [26] ^a^, [24] ^c^, Ireland
Feed conversion ratio	>4.0	>2.5, 3.0 and 4.0	>4.0	<3.0 and 6.0	[23] ^a^, [24] ^c^, Ireland
Final body weight	<2.0, 2.5 and 3.0	<2.5 and 3.0	-	-	[25] ^a^, [26] ^a^, [24] ^c^, Ireland
Kill out proportion	>2.0, 2.5 and 3.0	>3.0	-	-	[25] ^a^, [26] ^a^, [23] ^a^, Ireland
Live weight	<3.0	-	-	-	[25] ^a^, Ireland
**Physiology**					
Mean pre-ACTH cortisol concentration	<3.0	-	-	-	[25] ^a^, Ireland
Peak post-ACTH cortisol concentrations	<3.0	-	-	-	[25] ^a^, Ireland
Plasma NEFA concentrations	<3.0	-	-	-	[25] ^a^, Ireland

^a^ Age not provided; ^b^ <1 year; ^c^ 1–2 years.

**Table 6:** Overview of the effect of shade on beef cattle behavior, productivity, product quality and physiology. Studies are reported in the same language as they were presented by original study authors. Inclusion of significant results was determined at *p* < 0.05.

Metric Evaluated	Shade	Citation, Location
**Behavior**		
Feeding		
Proportion	>NS ^a^	[28] ^c^, South Africa
Mean panting scores	<NS	[29] ^d^, [28] ^c^, Australia, South Africa
**Productivity**		
ADG	>NS	[29] ^d^, Australia
DMI	>NS	[30] ^b^, [29] ^d^, [31] ^b^, USA, Australia
Final live weights	>NS	[31] ^b^, [29] ^d^, [28] ^c^, USA, Australia, South Africa
G:F	>NS	[29] ^d^, Australia
Hip height	>NS	[29] ^d^, Australia
**Product quality**		
Dark cutting carcasses	<NS	[31] ^b^, USA
Dressing percentage	>NS; <NS	[30] ^b^, [29] ^d^, USA, Australia
HCW	>NS	[29] ^d^, [28] ^c^, Australia, South Africa
USDA yield grade	>NS	[31] ^b^, USA
**Physiology**		
Lymphocytes (%)	>NS	[31] ^b^, USA
Neutrophils (%)	<NS	[31] ^b^, USA
Neutrophil: Lymphocyte ratio	<NS	[31] ^b^, USA
Respiration rate	<NS	[31] ^b^, [32] ^b^, USA

^a^ No shade (NS); ^b^ Age not provided; ^c^ <1 year; ^d^ 1–2 years.

**Table 7:** Overview of the effect of enrichment devices on beef cattle behavior, productivity and physiology. Enrichments are reported in the same language as they were presented by original study authors. Inclusion of significant results was determined at *p* < 0.05.

Metric Evaluated	Enrichments			Roofing	Ventilation	Citation, Location
	Brush and Log (BL)	Milk-Scent Releasing Device (MD)	Rubbing Devices (RD)	Modified Roof	Ceiling Van	
**Behavior**						
Abnormal breathing	-	-	-	-	<CON ^a^	[33] ^c^, Italy
Eating						
Duration	>CON	-	-	-	-	[34] ^d^, Japan
Enrichment use						
Frequency	-	>LD ^b^	>MD and LD	-	-	[35] ^e^, USA
Duration	-	-	>MD and LD	-	-	[35] ^e^, USA
Mounting						
Frequency	-	-	-	-	>CON	[33] ^c^, Italy
**Productivity**						
ADG	-	-	-	>CON	-	[36] ^f^, Thailand
**Physiology**						
Hygiene score	-	-	-	-	<CON	[33] ^c^, Italy
Rectal temperature	-	-	-	<CON	-	[36] ^f^, Thailand

^a^ Control (CON); ^b^ Lavender-scent releasing device; ^c^ Age not provided; ^d^ <1 year; ^e^ 1–2 years; ^f^ 2–3 years.

## Supplementary Materials

The following are available online at https://www.mdpi.com/2076-2615/10/4/565/s1, Table S1: Population parameters of the studies evaluated. Shown are the descriptions or values provided by the original authors of the articles these studies were chosen from, Table S2: Overview of the effect of different flooring types on beef cattle behavior, productivity, product quality, physiology and health. Flooring treatments are reported in the same language as they were presented by original study authors. Inclusion of significant results was determined at *p* < 0.05.
