# Supplementary material for: A Scoping Review: The Impact of Housing Systems and Environmental Features on Beef Cattle Welfare
Source: Animals (Basel). 2020 Mar 27;10(4):565. doi: 10.3390/ani10040565 (PMC7222360; doi:10.3390/ani10040565)
Supplement: Supplementary file 1 [file animals-10-00565-s001.pdf]

animals

Supplementary

Table 1. Population parameters of the studies evaluated. Shown are the descriptions or values provided by the original authors of the articles these studies were chosen from.

First Author (year); Country	Housing System	Production Stage	Experiment Length	Number of Animals (n)	Cattle Sex (cattle age)	Cattle Breed	Significant Results
Housing systems							
Andrighetto (1999); Italy [20]	Crates; Pens	Veal	102 days	34	Bull calves (<1 year)	Holstein	Yes
Blumetto (2017); Uruguay [19]	Pasture; Feedlot	Fattening	133 days	48	Steer (<1 year)	Holstein	Yes
Braghieri (2011); Italy [17]	Pasture; Feedlot	Fattening	6 months	12	Bull (<1 year)	Podolian	Yes
Ferrante (1999); Italy [16]	Crates; Pens	Veal	Not provided	24	Bull calves (<1 year)	Holstein	Yes
Johnson (2011); USA [18]	Hoop barn; Feedlot	Fattening	20 months	960	Steer (not provided)	Crossbred steers; predominately Angus	Yes
Tuomisto (2008); Finland [13]	Pasture; barn	Fattening	11 months	20	Bull (<1 year)	Hereford	Yes
Tuomisto (2015); Finland [15]	Pasture; feedlot	Fattening	62 days	29	Bull (1–2 years)	Hereford	Yes
Starvaggi Cucuzza (2014); Italy [21]	Feedlot	Fattening	Not provided	12	Bull (<1 year)	Piemontese	Yes
Space allowance							
Fisher (1997); Ireland [25]	Feedlot	Fattening	104 days	32	Heifer (not provided)	Charolais, Simmental and Hereford crossbred	Yes
Fisher (1997); Ireland [26]	Feedlot	Fattening	140 days	96	Heifer (not provided)	Simmental cross	Yes
Hickey (2003); Ireland [23]	Feedlot	Fattening	97 days	75	Steer (not provided)	Holstein	Yes
Keane (2017); Ireland [27]	Feedlot	Fattening	105 days	240	Heifer (1–2 years)	Crossbred	Yes
Keane (2018); Ireland [24]	Pasture; Feedlot	Fattening	105 days	120	Steer (1–2 years)	Charolais crossbred; Limousin crossbred	Yes
Ruis-Heutineck (2000); Netherlands [22]	Feedlot	Fattening	11 months	192	Bull (<1 year)	Piemontese x Black and White crossbred	Yes
Flooring							
Brscic (2015); Italy [38]	Feedlot	Fattening	9 months	326	Bull (not provided)	Charolais; Limousin	Yes
Brscic (2015); Italy [45]	Feedlot	Fattening	1 month	1440	Bull (not provided)	Charolais and French crossbred	Yes

	Feedlot	Fattening	4 months	1800	Bull (not provided)	Charolais and French crossbred	Yes
Cozzi (2005); Italy [46]	Feedlot	Fattening	7 months	1,338	Bull (not provided)	Charolais	Yes
Cozzi (2013); Italy [47]	Feedlot	Fattening	7 months	48	Bull (1–2 years)	Charolais and Aubrac crossbred	Yes
Earley (2015); Ireland [48]	Feedlot	Fattening	105 days	240	Heifer (1–2 years)	Continental cross; Holstein	Yes
Earley (2017); Ireland [49]	Feedlot	Fattening	7 months	360	Steer (not provided)	Continental crossbred	Yes
Elmore (2005); USA [41]	Feedlot	Fattening	3 months	48	Steer (<1 year)	Angus cross	Yes
Hickey (2003); Ireland [23]	Feedlot	Fattening	97 days	75	Steer (not provided)	Holstein	No
Keane (2015); Ireland [40]	Feedlot	Fattening	148 days	72	Bull (not provided)	Simmental crossbred	Yes
Keane (2017); Ireland [27]	Feedlot	Fattening	105 days	240	Heifer (1–2 years)	Crossbred	Yes
Platz (2007); Germany [39]	Feedlot	Fattening	1 year	18	Bull (<1 year)	Holstein and Fleckvieh crossbred	Yes
Ruis-Heutineck (2000); Netherlands [22]	Feedlot	Fattening	11 months	192	Bull (<1 year)	Piemontese x Black and White crossbred	Yes
Tessitore (2009); Italy [50]	Feedlot	Fattening	15 months	2,700	Bull (<1 year)	Charolais and French crossbred	Yes
Lowe (2001); Ireland [42]	Feedlot	Fattening	140 days	60	Steer (not provided)	Continental cross	Yes
	Feedlot	Fattening	142 days	80	Steer (not provided)	Continental cross	Yes
Yang (2017); South Korea [51]	Open barn	Fattening	6 months	12	Steer; Heifer (<1 year)	Hanwoo	Yes
Shade							
Blaine (2011); South Africa [28]	Feedlot	Fattening	36 days	146	Bull; Steer (<1 year)	Bonsmara crossbred	Yes
Bond (1975); USA [12]	Feedlot	Fattening	86 days	112	Bull (not provided)	Red Poll; Brown Swiss; Simmental and Hereford crossbred; Simmental and Angus crossbred; Limousin and Hereford crossbred; Limousin and Angus crossbred	No

	Feedlot	Fattening	84 days	120	Bull (not provided)	Simmental and Hereford crossbred; Simmental and Angus crossbred; Limousin and Hereford crossbred; Limousin and Angus crossbred	No
Brown-Bandl (2005); USA [32]	Feedlot	Fattening	37 days	8	Steer (not provided)	Crossbred (¼ Angus, ¼ Hereford; ¼ Pinzsauer; ¼ Red Poll)	Yes
Gaughan (2010); Australia [29]	Feedlot	Fattening	120 days	164	Steer (1–2 years)	Angus	Yes
Hagenmaier (2016); USA [30]	Feedlot	Fattening	1 month	1,395	Steer; Heifer (not provided)	Not provided; predominately black hided	Yes
Mitlöhner (2002); USA [31]	Feedlot	Fattening	121 days	168	Heifer (not provided)	Angus crossbred; Charolais crossbred	Yes
Van Iaer (2015); Belgium [14]	Pasture	Cow-calf	2 years	30	Cow (Ranged from 2 to 7 years)	Belgian Blue	No
Miscellaneous features							
Khongdee (2016); Thailand [36]	Barn	Fattening	157 days	10	Heifer (2–3 years)	Hindu Brazil-Brahman crosses	Yes
Magrin (2017); Italy [33]	Feedlot	Fattening	98 days	69	Bull (not provided)	Charolais	Yes
Ninomiya (2009); Japan [34]	Feedlot	Fattening	65 days	20	Steer (<1 year)	Japanese black; Japanese Shorthorn	Yes
Wilson (2002); USA [35]	Feedlot	Fattening	22 days	30	Heifer (1–2 years)	Charolais cross	Yes

Table 2. Overview of the effect of different flooring types on beef cattle behavior, productivity, product quality, physiology and health. Flooring treatments are reported in the same language as they were presented by original study authors. Inclusion of significant results was determined at $p < 0.05$.

	**Floor Types**								
	**Concrete**		**Rubber Mats**					**Natural Materials**	
**Metrics Evaluated**	**Flat Concrete Floor (FC)**	**Fully Slatted Concrete Floor (FSC)**	**Fully Slatted Rubber Mats (FSRM)**	**Perforated Rubber Mats (PRM)**	**Natural Rubber Structure (EasyFix)**	**Rubber Mat (Irish Custom Extenders; ICE)**	**Outwintering Pads (OP)**	**Sawdust (S)**	**Reference, Location**
Behavior									
Abnormal behaviors									
Duration	-	>FSRM	-	-	-	-	-	-	{22} [i], Netherlands
Abnormal lying down									
Frequency	-	>FSRM	-	-	-	-	-	-	{38} [h], Italy
Activity									
Duration	-	<FSRM	-	-	-	-	-	-	{38} [h], Italy
Agonistic interactions									
Duration	-	<DL [1]	-	-	-	-	-	-	{50} [i], Italy
Frequency	-	-	-	-	-	-	-	>RH [2] and WS[3]	{51} [i], South Korea
Avoidance from feedrack	-	<DL	-	-	-	-	-	-	{50} [i], Italy
Chasing									
Frequency	-	<FSRM	-	-	-	-	-	-	{38} [h], Italy
Cohesive interaction									
Duration	-	>DL	-	-	-	-	-	-	{50} [i], Italy
Drinking									
Duration	-	>EVA [4], OP and DUR[5]	-	-	>OP	-	<EVA and DUR	-	{49} [h], Ireland
Eating									
Duration	-	<DL, EVA, FSRM, OP and DUR	-	-	<DL, EVA, OP and DUR	<DL	>EVA and DUR	-	{48} [j], {49} [h], {38} [h], Ireland, Italy
Grooming									
Duration	-	<SRMPC [6]	-	-	-	>DL and EasyFix	-	-	{41} [i], {48} [j], USA, Ireland
Headbutting									
Frequency	-	<DL and FSRM	-	-	-	-	-	-	{48} [j], {38} [h], Ireland, Italy
Proportion	-	-	-	-	>FSC	-	-	-	{48} [j], Ireland
Inactive									
Proportion	-	>FSRM	-	-	-	-	-	-	{38} [h], Italy
Lying down attempts	-	>PRM	-	-	-	-	-	-	{47} [g], Italy
Lying									

Duration	-	>DL, FSRM, OP, PSRM[7] and PRM; <SRMPC	-	-	-	-	<EVA and DUR	-	{50} [i], {38} [h], {39} [i],{49} [h], {47} [j], {41} [i], Italy, Germany, Ireland, USA
Frequency	-	<FSRM and S	<S	-	-	-	-	-	{22} [i], Netherlands
Proportion	-	<ICE and S	-	-	>DL, EVA, FSC, OP and DUR	>DL	-	-	{48} [j], {27} [j], {49} [h], Ireland
Mounting									
Duration	-	<DL, FSRM, EasyFix and PRM	-	-	-	-	-	-	{45} [h], {50} [i], {38} [h], {48} [j], {47} [j], Italy, Ireland
Proportion	-	-	-	-	-	>DL and EasyFix	-	-	{48} [j], Ireland
Postural changes	-	<FSRM	>SRMPC	-	-	-	-	-	{41} [i], USA
Resting									
Proportion	-	>FSRM	-	-	-	-	-	-	{38} [h], Italy
Standing									
Duration	-	<DL	-	-	-	-	-	-	{50} [i], Italy
Unsuccessful lying event									
Frequency	-	>FSRM	-	-	-	-	-	-	{38} [h], Italy
Productivity									
Average daily gain	-	<FSRM, PRM and S	-	-	-	-	-	-	{38} [h], {40} [h], {47} [j], {27} [j], Italy, Ireland
Dry matter intake	-	<OP	-	-	<OP	-	>EVA and DUR	-	{49} [h], Ireland
Feed conversion ratio	-	< FSRM and S	-	-	-	-	-	-	{40} [h], {27} [j], Ireland
Final live weight	-	<FSRM, PRM and S	>S	-	-	-	-	>RH	{38, 27, 22, 51} [i], South Korea
Live weight gain	-	<EVA, FSRM, OP and DUR	>S	-	<EVA, OP and DUR	-	>EVA and DUR	-	{49} [h], {40} [h], {22} [i], Ireland, Netherlands
Product quality									
Carcass gain	-	<FSRM	-	-	-	-	-	-	{40} [h], Ireland
Carcass weight	-	<S	-	-	-	-	-	-	{27} [j], Ireland
Hide weight	-	<FSRM and S	-	-	-	-	-	-	{40} [h], {27} [j], Ireland
Kidney and channel fat weights	-	<FSRM	-	-	-	-	-	-	{40} [h], Ireland
Physiology									
Total serum protein	-	>S	-	-	-	-	-	-	{27} [j], Ireland
Health									
Bursitis prevalence	-	>DL and FSRM	-	-	-	-	-	-	{45} [h], {38} [h], Italy

Early culling (%)	-	>DL	-	-	-	-	-	-	{45} [h], Italy
Gait scores	-	>FSRM	<SRMPC	-	-	-	-	-	{32} [i], USA
Hairless patch prevalence	-	>DL	-	-	-	-	-	-	{45} [h], Italy
Hock swelling scores	-	>FSRM	<SRMPC	-	-	-	-	-	{41} [i], USA
Hoof erosion	-	<ICE	-	-	>DL and FSC	>DL	-	-	{48} [j], Ireland
Hoof lesion prevalence	-	<DL, EVA, FSRM, EasyFix, DUR and ICE	>S	-	-	-	-	-	{48} [j], {49} [h], {40} [h], Ireland
Hoof overgrowth prevalence	-	<FSRM, EasyFix and ICE	-	-	>DL	-	-	-	{38} [h], {48] [j], Italy, Ireland
Hygiene score	>PSF and S	<DL, OP and SRMPC; >ICE	<SRMPC; >S	>S and SS [8]	<OP	<DL; >EasyFix	>EVA and DUR	-	{46} [h], {42} [h], {48} [j], {49} [h], {41} [i], Italy, Ireland, USA
Knee swelling	-	>FSRM	<SRMPC	-	-	-	-	-	{41} [i], USA
Lesions / swelling prevalence	-	>DL, FSRM and PSRM; <SRMPC	<SRMPC	-	-	-	-	-	{45} [h], {39} [i], {41} [i], Italy, Germany, USA
Nasal discharge prevalence	-	<DL	-	-	-	-	-	-	{45} [h], Italy
Pen cleanliness	-	Cleaner than FSRM and SRMPC	Cleaner than SRMPC	-	-	-	-	-	{41} [i], USA
Severe lesions prevalence	-	>FSRM	<S	-	-	-	-	-	{22} [i], Netherlands
Slipping events	-	>PC and PRM	-	-	-	-	-	-	{47} [g], Italy
Treatments for locomotor disorders Proportion	-	> FSRM	-	-	-	-	-	-	{38} [h], Italy

[1] Deep litter; [2] Rice husks; [3] Wood shavings; [4] Foam structure rubber mat; [5] Rubber mat (Durapak); [6] Solid rubber mat—partial cover; [7] Partially slatted rubber mats; [8] Strips of rubber secured to slats; [h] Age not provided; [i] <1 year; [j] 1–2 years.

Supplementary References

1. Angrecka, S.; Herbut, P.; Nawalany, G.; Sokołowski, P. The impact of localization and barn type on insolation of sidewall stalls during summer. *J. Ecol. Eng.* **2017**, *18*, 60–66, doi:10.12911/22998993/74398.
2. Angrecka, S.; Herbut, P. Impact of barn orientation on insolation and temperature of stalls surface. *Ann. Anim. Sci.* **2016**, *16*, 887–896, doi:10.1515/aoas-2015-0096.

3. Scientific Committee on Animal Health and Animal Welfare (SCAHAW): The welfare of cattle kept for beef production. Available online: http://ec.europa.eu/food/fs/sc/scah/out54_en.pdf (accessed on 19 October 2018).
4. European Food Safety Authority (EFSA) Panel on Animal Health and Welfare. Scientific opinion on the welfare of cattle kept for beef production and the welfare in intensive calf farming systems. *EFSA J.* **2012**, *10*, 1–165.
5. European Food Safety Authority (EFSA). Scientific Opinion of the Panel on Animal Health and Welfare (AHAW Panel) on a request from the European Commission on the risks of poor welfare in intensive calf farming systems. An update of the Scientific Veterinary Committee Report on the Welfare of Calves. *EFSA J.* **2006**, *366*, 1–36.
6. Lonne Ingvartsen, K.; Refsgaard Anderson, H. Space allowance and type of housing for growing cattle: A review of performance and possible relation to neuroendocrine function. *Acta Agric. Scand*. **1993**, *43*, 65–80, doi:10.1080/09064709309410147.
7. O'Connor, A.M.; Anderson, K.M.; Goodell, C.K.; Sargeant, J.M. Conducting systematic reviews of intervention questions I: Writing the review protocol, formulating the question and searching the literature. *Zoonoses Public Health* **2014**, *61*, 28–38, doi:10.1111/zph.12125.
8. Sargeant, J.M.; O'Connor, A.M. Conducting systematic reviews of intervention questions II: Relevance screening, data extraction, assessing risk of bias, presenting the results and interpreting the findings. *Zoonoses Public Health.* **2014**, *61*, 39–51, doi:10.1111/zph.12124.
9. Broom, D.M. Coping, stress and welfare. In *Coping with Challenge: Welfare in Animals including Humans*; Broom, D.M., Ed.; Dahlem University Press: Berlin, Germany, 2001.
10. Ouzzani, M.; Hammady, H.; Fedorowicz, Z.; Elmagarmid, A. Rayyan—A web and mobile app for systematic reviews. *Syst. Rev.* **2016**, *5*, 210, doi:10.1186/s13643-016-0384-4.
11. Higgins, J.P.T.; Altman, D.G.; Gøtzsche, P.C.; Jüni, P.; Moher, D.; Oxman, A.D.; Savović, J.; Schulz, K.F.; Weeks, L.; Sterne, J.A.C.; Cochrane Bias Methods Group and Cochrane Statistical Methods Group. The Cochrane Collaboration's tool for assessing risk of bias in randomised trials. *BMJ* **2011**, *343*, d5928, doi:10.1136/bmj.d5928.
12. Bond, T.E.; Laster, D.B. Influence of shading on production of Midwest feedlot cattle. *Trans. ASAE* **1975**, *18*, 957–959.
13. Tuomisto, L.; Ahola, L.; Martiskainen, P.; Kauppinen, R.; Huuskonen, A. Comparison of time budgets of growing Hereford bulls in an uninsulated barn and in extensive forest paddocks. *Livest. Sci.* **2008**, *118*, 44–52, doi:10.1016/j.livsci.2008.01.011.
14. Van Iaer, E.; Moons, C.P.H.; Ampe, B.; Sonck, B.; Vandaele, L.; De Campeneere, S.; Tuyttens, F.A.M. Effect of summer conditions and shade on behavioural indicators of thermal discomfort in Holstein dairy and Belgian Blue beef cattle on pasture. *Animal* **2015**, *9*, 1536–1546, doi:10.1017/S1751731115000804.
15. Tuomisto, L.; Mononen, J.; Martiskainen, P.; Ahola, L.; Huuskonen, A. Time budgets of finishing bulls housed in an uninsulated barn or at pasture. *Agr. Food Sci.* **2015**, *24*, 173–182.
16. Ferrante, V.; Canali, E.; Verga, M.; Mattiello, S.; Menti, F.; Gottardo, F. Veal calves rearing: Behavioural, physiological and pathological indicators. In Proceedings of the A.S.P.A. XII Congress, Piacenza, Italy, 21–24 June 1999; pp. 575–577.
17. Braghieri, A.; Pacelli, C.; De Rosa, G.; Girolami, A.; De Palo, P.; Napolitano, F. Podolian beef production on pasture and in confinement. *Animal* **2011**, *5*, 927–937, doi:10.1017/S1751731110002685.
18. Johnson, A.; Lonergan, S.M.; Busby, W.D.; Shouse, S.C.; Maxwell, D.L. Comparison of steer behavior when housed in a deep-bedded hoop barn versus an open feedlot with shelter. *J. Anim. Sci.* **2011**, *89*, 1893, doi:10.2527/jas.2010-2877.
19. Blumetto, O.; Ruggia, A.; Morales Pyñeyrúa, J.T.; Villagrá García, A. Social behavior and productive and stress parameters in Holstein steers fattened in three contrasting production systems. *J. Agric. Sci.* **2017**, *9*, 54–64, doi:10.5539/jas.v9n5p54.
20. Andrighetto, I.; Gottardo, F.; Andreoli, D.; Cozzi, G. Effect of type of housing on veal calf growth performance, behaviour and meat quality. *Livest. Prod. Sci.* **1999**, *57*, 137–145.
21. Starvaggi Cucuzza, L.; Riondato, F.; Macchi, E.; Bellino, C.; Franco, G.; Biolatti, B.; Cannizzo, F.T. *Res. Vet. Sci.* **2014**, *97*, 465–470, doi:10.1016/j.rbsc.2014.08.002.
22. Ruis-Heutinck, L.F.M.; Smits, M.C.J.; Smits, A.C.; Heeres, J.J. Effects of floor type and floor area on behavior and carpal joint lesions in beef bulls. *Improv. Health Welf. Anim. Prod.* **2000**, *102*, 29–36.

23. Hickey, M.C.; Earley, B.; Fisher, A.D. The effect of floor type and space allowance on welfare indicators of finishing steers. *Ir. J. Agric. Food Res.* **2003**, *42*, 89–100.
24. Keane, M.P.; McGee, M.; O'Riordan, E.G.; Kelly, A.K.; Earley, B. Performance and welfare of steers housed on concrete slatted floors at fixed and dynamic (allometric based) space allowances. *J. Anim. Sci.* **2018**, *96*, 880–889, doi:10.1093/jas/sky007.
25. Fisher, A.D.; Crowe, M.A.; Prendiville, D.J.; Enright, W.J. Indoor space allowance: Effects on growth, behaviour, adrenal and immune responses of finishing beef heifers. *J. Anim. Sci.* **1997**, *64*, 53–62.
26. Fisher, A.D.; Crowe, M.A.; O'Kiely, P.; Enright, W.J. Growth, behaviour, adrenal and immune responses of finishing beef heifers housed on slatted floors at 1.5, 2.0, 2.5 or 3.0 m^2 space allowance. *Livest. Prod. Sci.* **1997**, *51*, 245–254.
27. Keane, M.P.; McGee, M.; O'Riordan, E.G.O.; Kelly, A.K.; Earley, B. Effect of space allowance and floor type on performance, welfare and physiological measurements of finishing beef heifers. *Animal* **2017**, *11*, 2285–2294, doi:10.1017/S1751731117001288.
28. Blaine, K.L.; Nsahlai, I.V. The effects of shade on performance, carcass classes and behaviour of heat-stressed feedlot cattle at the finisher phase. *Trop. Anim. Health Prod.* **2010**, *43*, 609–615, doi:10.1007/s11250-010-9740-x.
29. Gaughan, J.B.; Bonner, S.; Loxton, I.; Mader, T.L.; Lisle, A.; Lawrence, R. Effect of shade on body temperature and performance of feedlot steers. *J. Anim. Sci.* **2010**, *88*, 4056–4067, doi:10.2527/jas.2010-2987.
30. Hagenmaier, J.A.; Reinhardt, C.D.; Bartle, S.J.; Thomson, D.U. Effect of shade on animal welfare, growth performance, and carcass characteristics in large pens of beef cattle fed a beta agonist in a commercial feedlot. *J. Anim. Sci.* **2016**, *94*, 5064–5076, doi:10.2527/jas2016-0935.
31. Mitlöhner, F.M.; Galyean, M.L.; McGlone, J.J. Shade effects on performance, carcass traits, physiology, and behavior of heat-stressed feedlot heifers. *J. Anim. Sci.* **2002**, *80*, 2043–2050.
32. Brown-Brandl, T.M.; Eigenberg, R.A.; Nienaber, J.A.; Hahn, G.L. Dynamic response indicators of heat stress in shaded and non-shaded feedlot cattle, part 1: Analyses of indicators. *Biosyst. Eng.* **2005**, *90*, 451–462.
33. Brscic, M.; Gottardo, F.; Tessitore, E.; Guzzo, L.; Ricci, R.; Cozzi, G. Assessment of welfare of finishing beef cattle kept on different types of floor after short- or long-term housing. *Animal* **2015**, *9*, 1053–1058, doi:10.1017/S1751731115000245.
34. Ninomiya, S.; Sato, S. Effects of "Five Freedoms" environmental enrichment on the welfare of calves reared indoors. *J. Anim. Sci.* **2009**, *80*, 347–351, doi:10.1111/j.1740-0929.2009.00627.x.
35. Wilson, S.C.; Mitlöhner, F.M.; Morrow-Tesch, J.; Dailey, J.W.; McGlone, J.J. An assessment of several potential enrichment devices for feedlot cattle. *Appl. Anim. Beh. Sci.* **2002**, *76*, 259–265.
36. Cozzi, G.; Ricci, R.; Dorigo, M.; Zanet, D. Growth performance, cleanliness and lameness of finishing Charolais bulls housed in littered pens of different designs. *Ital. J. Anim. Sci.* **2005**, *4*, 251–253, doi:10.4081/ijas.2005.2s.251.
37. Da Costa, B.R.; Beckett, B.; Diaz, A.; Resta, N.M.; Johnston, B.C.; Egger, M.; Jüni, P; Armijo-Olivo, S. Effect of standardized training on the reliability of the Cochrane risk of bias assessment tool: a prospective study. *Syst. Rev.* **2017**, *6(44)*, https://doi.org/10.1186/s13643-017-0441-7.
38. Brscic, M.; Ricci, R.; Prevedello, P.; Lonardi, C.; De. Nardi, R.; Contiero, B.; Gottardo, F.; Cozzi, G. Synthetic rubber surface as an alternative to concrete to improve welfare and performance of finishing beef cattle reared on fully slatted flooring. *Animal* **2015**, *9*, 1386–1392, doi:10.1017/S1751731115000592.
39. Platz, S.; Ahrens, F.; Bahrs, E.; Nüske, S.; Erhard, M.H. Association between floor type and behaviour, skin lesions, and claw dimensions in group-housed fattening bulls. *Prev. Vet. Med.* **2007**, *80*, 209–221, doi:10.1016/j.prevetmed.2007.02.007.
40. Keane, M.P.; McGee, M.; O'Riordan, E.G.; Kelly, A.K.; Earley, B. Effect of floor type on hoof lesions, dirt scores, immune response and production of beef bulls. *Livest. Sci.* **2015**, *180*, 220–225, doi:10.1016/j.livsci .2015.08.002.
41. Elmore, M.R.P.; Elischer, M.F.; Claeys, M.C.; Pajor, E.A. The effects of different flooring types on the behavior, health and welfare of finishing beef steers. *J. Anim. Sci.* **2005**, *93*, 1258–1266, doi:10.2527/jas2014-8399.
42. Lowe, D.E.; Steen, R.W.J.; Beattie, V.E.; Moss, B.W. The effects of floor type systems on the performance, cleanliness, carcass composition and meat quality of housed finishing beef cattle. *Livest. Prod. Sci.* **2001**, *69*, 33–42.

43. Institute of Medicine (U.S.). Committee on Standards for Systematic Reviews of Comparative Effectiveness Research. In *Finding What Works in Health Care: Standards for Systematic Reviews*; National Academies Press: Washington, DC, USA, 2011.
44. Rethlefsen, M.L.; Farrell, A.M.; Osterhaus Trzasko, L.C.; Brigham, T.J. Librarian co-authors correlated with higher quality reported search strategies in general internal medicine systematic reviews. *J. Clin. Epidemiol.* **2015**, *68*, 617–626, doi:10.1016/j.jclinepi.2014.11.025.
45. Brscic, M.; Gottardo, F.; Tessitore, E.; Guzzo, L.; Ricci, R.; Cozzi, G. Assessment of welfare of finishing beef cattle kept on different types of floor after short- or long-term housing. *Animal* **2015**, *9*, 1053–1058, doi:10.1017/S1751731115000245.
46. Cozzi, G.; Ricci, R.; Dorigo, M.; Zanet, D. Growth performance, cleanliness and lameness of finishing Charolais bulls housed in littered pens of different designs. *Ital. J. Anim. Sci.* **2005**, *4*, 251–253, doi:10.4081/ijas.2005.2s.251.
47. Cozzi, G.; Tessitore, E.; Contiero, B.; Ricci, R.; Gottardo, F.; Brscic, M. Alternative solutions to the concrete fully-slatted floor for the housing of finishing beef cattle: Effects on growth performance, health of the locomotor system and behaviour. *Vet. J.* **2013**, *197*, 211–215, doi:10.1016/j.tvjl.2013.03.001.
48. Earley, B.; McNamara, J.D.; Jerrrams, S.J.; O-Riordan, E.G. Effect of concrete slats, three mat types and out-wintering pads on performance and welfare of finishing beef steers. *Acta Vet. Scand.* **2017**, *59*, 1–14, doi:10.1186/s13028-017-0302-3.
49. Khongdee, T. Effects of roof modifications on growth performance and physiological changes of crossbred beef heifers *(Bos indicus)*. Songklanakarin. *J. Sci. Technol*. **2016**, *38*, 183–188.
50. Tessitore, E.; Boukha, A.; Guzzo, L.; Cozzi, G. Differences in behavior, health status and productive performance of beef young bulls housed on different type of floor and assessed in two fattening phases. *Ital. J. Anim. Sci.* **2009**, *8*, 39–57.
51. Yang, K.Y.; Park, K.; Ha, J.J.; Gotoh, T.; Song, Y.H. Effect of pen floor material on the behavior of Hanwoo cattle. *J. Fac. Agr. Kyrushu Univ.* **2017**, *1*, 99–103.

© 2020 by the authors. Submitted for possible open access publication under the terms and conditions of the Creative Commons Attribution (CC BY) license (http://creativecommons.org/licenses/by/4.0/).
